# Aberrant Neurogliovascular Unit Dynamics in Cerebral Small Vessel Disease: A Rheological Clue to Vascular Parkinsonism

**DOI:** 10.3390/pharmaceutics13081207

**Published:** 2021-08-05

**Authors:** Che Mohd Nasril Che Mohd Nassir, Thenmoly Damodaran, Siti R. Yusof, Anwar Norazit, Geetha Chilla, Isaac Huen, Bhanu Prakash K. N., Norlinah Mohamed Ibrahim, Muzaimi Mustapha

**Affiliations:** 1 Department of Neurosciences, School of Medical Sciences, Universiti Sains Malaysia, Kubang Kerian 16150, Kelantan, Malaysia; nasrilche123@gmail.com; 2 Centre for Drug Research, Universiti Sains Malaysia, Minden 11800, Penang, Malaysia; dthenmoly@gmail.com (T.D.); sryusof@usm.my (S.R.Y.); 3 Department of Biomedical Science, Faculty of Medicine, University of Malaya, Kuala Lumpur 50603, Selangor, Malaysia; anwar.norazit@um.edu.my; 4 A*STAR Institute of Bioengineering and Bioimaging, Helios, 11 Biopolis Way, Singapore 138667, Singapore; venkata_naga_geetha@ibb.a-star.edu.sg (G.C.); isaac_huen@ibb.a-star.edu.sg (I.H.); bhanu@ibb.a-star.edu.sg (B.P.K.N.); 5 Department of Medicine, Faculty of Medicine, Universiti Kebangsaan Malaysia, Kuala Lumpur 56000, Selangor, Malaysia; norlinah@ppukm.ukm.edu.my; 6 Hospital Universiti Sains Malaysia, Jalan Raja Perempuan Zainab II, Kubang Kerian 16150, Kelantan, Malaysia

**Keywords:** cerebral small vessel disease, vascular parkinsonism, homeostasis, neurogliovascular unit, glymphatic system

## Abstract

The distinctive anatomical assemble and functionally discrete multicellular cerebrovasculature dynamics confer varying rheological and blood–brain barrier permeabilities to preserve the integrity of cerebral white matter and its neural microenvironment. This homeostasis intricately involves the glymphatic system that manages the flow of interstitial solutes, metabolic waste, and clearance through the venous circulation. As a physiologically integrated neurogliovascular unit (NGVU) serving a particularly vulnerable cerebral white matter (from hypoxia, metabolic insults, infection, and inflammation), a likely insidious process over a lifetime could inflict microenvironment damages that may lead to pathological conditions. Two such conditions, cerebral small vessel disease (CSVD) and vascular parkinsonism (VaP), with poorly understood pathomechanisms, are frequently linked to this brain-wide NGVU. VaP is widely regarded as an atypical parkinsonism, described by cardinal motor manifestations and the presence of cerebrovascular disease, particularly white matter hyperintensities (WMHs) in the basal ganglia and subcortical region. WMHs, in turn, are a recognised imaging spectrum of CSVD manifestations, and in relation to disrupted NGVU, also include enlarged perivascular spaces. Here, in this narrative review, we present and discuss on recent findings that argue for plausible clues between CSVD and VaP by focusing on aberrant multicellular dynamics of a unique integrated NGVU—a crossroad of the immune–vascular–nervous system—which may also extend fresher insights into the elusive interplay between cerebral microvasculature and neurodegeneration, and the potential therapeutic targets.

## 1. Introduction

The distinctive anatomical assemble and functionally discrete multicellular cerebrovasculature dynamics confer varying rheological and blood–brain barrier (BBB) permeabilities to preserve the integrity of cerebral white matter and its neural microenvironment. This homeostasis intricately involves the neurogliovascular unit (NGVU) and glymphatic system that manages the flow of cerebrospinal fluid (CSF), interstitial fluids (ISF), metabolic waste, and clearance through the venous circulation. NGVU refers to the integration of neuronal structures, glial cells (including the microglial), and vasculature (i.e., capillaries, arteries, and/or arterioles) governed by the astrocytes [1,2]. The neurotoxic soluble waste products are continuously released from the NGVU into the ISF space due to the brain’s high metabolic rate [3] and the glymphatic pathway is considered a major route for the drainage of those metabolites.

As a physiologically integrated NGVU and glymphatic system serving a particularly vulnerable cerebral white matter (from hypoxia, metabolic insults, infection, and inflammation), a likely insidious lifetime process could inflict microenvironment damages that may lead to pathological conditions. Two such conditions, cerebral small vessel disease (CSVD) and vascular (or arteriosclerotic) parkinsonism (VaP), with poorly understood pathomechanisms are linked to this brain-wide NGVU. CSVD is a spectrum of a chronic, progressive disorder affecting the cerebral microvasculature (or microcirculation) involving small (or micro, sizes 50–400 µm) penetrating arteries (chiefly of middle cerebral artery tributaries), arterioles, capillaries, and small veins (or venules) that penetrate and supply the white matter and deep grey matter of the brain subcortical region [4,5]. On the other hand, VaP, which accounts for about 4.4 to 12% of parkinsonism [6], is phenotypically characterised by symmetrical lower-body parkinsonism and vascular lesions, particularly white matter hyperintensities (WMHs) or multi-infarct in the basal ganglia and subcortical region with predilection in the elderly (>65 years) and in males [7,8,9]. The WMHs, in turn, are a recognised imaging spectrum of CSVD manifestations, and in relation to a disrupted NGVU, also include enlarged perivascular spaces (ePVS). 

Therefore, in this narrative review, we present and discuss recent findings that argue for plausible clues between CSVD and VaP by focusing on aberrant multicellular dynamics of a unique integrated NGVU and glymphatic system—a crossroad of the immune–vascular–nervous system—which may also extend fresher insights into the elusive interplay between cerebral microvasculature and neurodegeneration, and the potential therapeutic targets.

### Literature Search Strategy

The methods used for the literature search included the use of online databases and search engines for a specific keywords combination. Numerous online databases utilised include Google Scholar, Science Direct, PubMed, Medline, Wiley Online Library, and the ISI Web of Knowledge. Articles such as systematics reviews, meta-analyses, narrative reviews, research papers, randomized clinical trials and cohort studies were included, and restricted to the English version only. The literature search was based on the following keywords that progressed incrementally from simple (single keywords) to compound (combined keywords): cerebral small vessel disease, vascular parkinsonism, homeostasis, neurogliovascular unit, glymphatic system, and aging. From the purposive selection of articles retrieved, further references were then probed by a manual search among the cited references for a discursive analysis and synthesis from one topic to the next as deliberated in this narrative review.

## 2. Overview of the Neurogliovascular Unit

The long-accepted view, that information processing networks in the brain are mainly governed by the brain’s neurons, is being expanded with growing bodies of evidence that other brain cells are equally crucial in maintaining the homeostasis and physiology of the brain activity [10,11]. These other neural cells include the glial cells that constitute about 50% of the brain’s volume, although the approximate numbers and ratio of neuron to glial in the brain differ across species [12,13]. Moreover, due to evolving developments in glial research, studies have found that glial cells extensively contribute to the brain structure and function through the modulation of neurotransmitter and synaptic activity, induction, and maintenance of barrier properties of the brain endothelium that forms the BBB, potassium ion (K^+^) movement (or K^+^ siphoning), and global brain’s computation [14,15,16,17,18].

**Figure 1:**
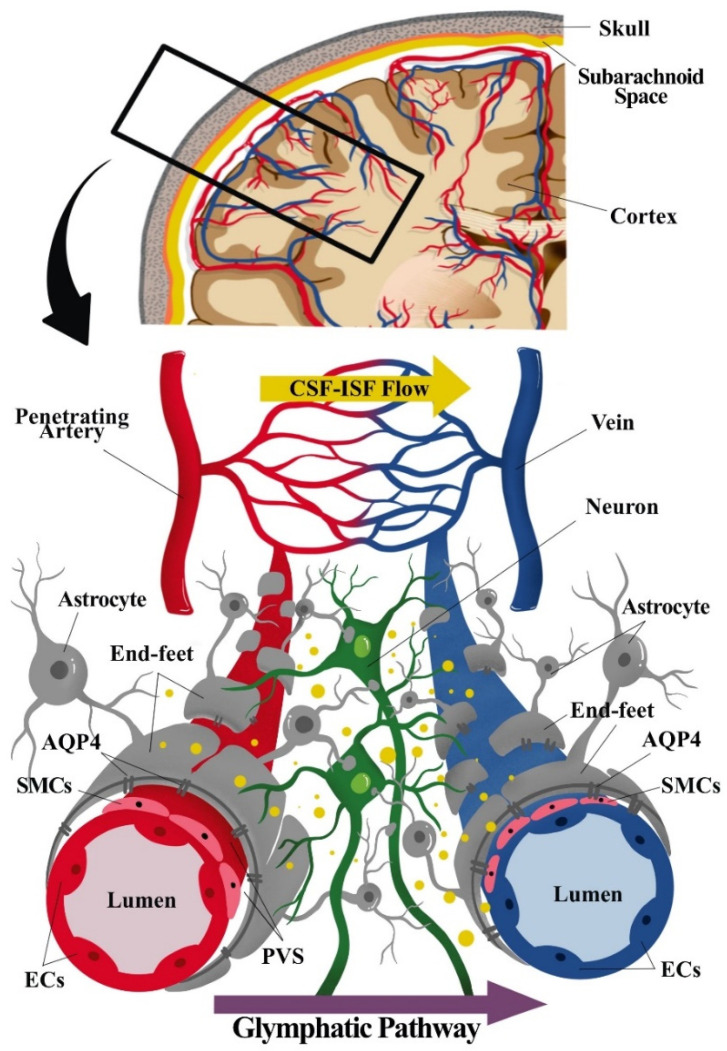
Component of the neurogliovascular unit (NGVU) and glymphatic system. The NGVU includes the neuronal cells, glial cells, and the vasculature (arteries and veins), including the smooth muscle cells (SMCs) and the pericytes (not shown in the figure) surrounding the vascular endothelial cells (ECs). Whereby, the glymphatic system consists mainly of astrocytes and astrocytic end-feet sheathing the vasculature. The transport of waste product through cerebrospinal fluid (CSF) from arteries passing through astrocytic aquaporin pore-4 (AQP4) into the interstitial fluid (ISF), hence, mixing of CSF and ISF. The waste solutes and/or by-products flow (indicated as yellow dots in the figure) following the glymphatic pathway to be absorbed for further waste clearance system.

The BBB is at the level of cerebral microvascular endothelium that forms the microvascular wall, of which properties are induced by the associating cells, particularly astrocytes [19]. Astrocytes, among others, are the type of glial cells that modulate the ongoing neurotransmission process through peri-synaptic processes. These astrocytic processes wrap around the cerebral vasculature [20,21]; hence, facilitating the vasoactive signal [22] (see Figure 1). Moreover, emerging experimental data supports that vascular vasomotion may also influence the neuronal activities termed as “reverse influence”, and the disturbances of these neurons-to-glial-to-vascular or vascular-to-neurons dynamics may lead to cerebrovascular and neurodegenerative disease [23,24]. These advancements in cerebrovascular and glial research highlight the importance of the dynamic interactions between neuronal cells, glial cells (governed by astrocytes), and the cerebral vascular systems (including microvessel) in health and disease states, which led to the term neurogliovascular unit (NGVU) being introduced [1,2].

### 2.1. Neurogliovascular Unit: Structure, Function, and Metabolic Coupling

The NGVU signalling and/or dynamics are crucial for the sustainability of the brain structure and functions, and any disturbances to these systems contribute to numerous neuropathologies. The cellular components of the NGVU include specialized brain capillary endothelial cells (ECs), which form the vascular barrier with restrictive tight junctions, surrounded by pericytes, vascular smooth muscles cells (vSMCs), neurons, and glial cells [2,19] (see Figure 1). The NGVU also involves the perivascular space (PVS) or Virchow–Robin space between the endothelium and basement membranes of the brain parenchyma at the pre- and postcapillary levels. The PVS, together with the glymphatic system, aids in fluids transport for the removal of waste products and biologically active molecules [25,26].

Functionally, the NGVU modulates the metabolic demands together with the cerebral blood flow (cBF), in addition to the adenosine-mediated vasodilation following metabolic activities and EC-derived nitric oxide (NO) signalling [27]. Meanwhile, as aforementioned, astrocytes integrate and convey neuronal activities to vasoactive signals [28]. For example, during a synaptic transmission the concentration of calcium ion (Ca^2+^) increases in the peri-synaptic end-feet processes; hence, propagating through the astrocytic processes via activated phospholipase A_2_. This, in turn, initiates the synthesis of vasoactive arachidonic acid (AA) metabolites in the end-feet surrounding vascular capillaries and arterioles by epoxygenases and cyclooxygenases [29,30]. Subsequently, the AA metabolites formed may diffuse into the neighbouring vSMCs or pericytes of the vascular wall whilst ω-hydroxylase metabolizes the AA metabolites to 20-hydroxyeicosatetraenoic (20-HETE). Depending on the concentration of Ca^2+^ and local oxygen (O_2_) at the end-feet, the synthesis of either 20-HETE or prostaglandin E_2_ may predominantly lead to vascular constriction or dilation, respectively [28,31]. On the other hand, the astrocytic Ca^2+^ signalling may also be triggered by the changes in the extracellular Ca^2+^ concentration [32]. Following this, the K^+^ ions are released from the astrocytes onto the vSMCs, causing hyperpolarisation of the vSMCs by opening large-conductance Ca^2+^-activated K^+^ channels that are largely expressed on the perivascular end-feet plasmalemma. This process is known as smooth muscle K^+^-dependent relaxation or “K^+^ siphoning” [33,34], i.e., the movement of the K^+^ ion inside the NGVU, from the neurons, through the glial cell (astrocytes), to the vessels. The amount of K^+^ ion released determines the vessel dynamics, in this case a modest increment of Ca^2+^ at the astrocytic end-feet that induces dilation, whilst a higher increment induces the constriction [27]. Thus, K^+^ ion is considered a mediator of metabolic coupling between neuronal activity and cBF. 

Furthermore, Ca^2+^ increments in neurons and/or interneurons also contribute to the regulation of local cBF. One example is the change in vascular dynamics mediated by the endothelium-dependent relaxation response to acetylcholine (ACh) via the NO pathway. In this case, ACh leads to endothelium vasodilation by a stimulation of the production of NO by endothelial NO synthase (eNOS) activation. The diffusion of NO to the vSMCs and/or pericytes induces vasorelaxation by the activation of the soluble guanylate cyclase and the subsequent production of cyclic guanosine monophosphate (cGMP) [35]. In short, the relative changes in the cBF in response to neuronal activity is a complex multisystem signalling/pathway involving various vasoactive mediators in the NGVU (i.e., the neurons, glial cells (astrocytes), and vasculature). However, different brain regions may have different NGVU functional pathways. For example, interneurons activated by glutamatergic collaterals and the complex topographical relationship between the cellular compartments of NGVU may account for these disparities [28,36]. Immediate, short lasting and sustained vasodilatory responses may also differ depending on the mediators and pathways involved [37]; hence, any aberrancy in these multisystem dynamics may lead to differing neurological syndromes though they share similar fundamental and/or overlapping rheological features. 

### 2.2. The Glymphatic System and NGVU

As discussed, a sub-component of the NGVU such as the CSF-filled PVS or Virchow–Robin space surrounding the cerebral perforating arteries and veins is currently subject to active research following the discovery of the glymphatic (or clearance) system (i.e., the removal and transport of waste products and biologically active molecules), whereby PVS is the fundamental node [38]. The term glymphatic refers to the “glia + lymphatic” pathway or brain’s “front end” of waste clearance, named after the primary role of glial cells and the similarities to the authentic extracranial lymphatic system [39,40]. Generally, the glymphatic pathway includes a PVS network for CSF transport that is connected to a downstream authentic lymphatic system associated with the dura meninges, cranial nerves, and large vessels exiting the skull [40,41]. It is suggested that neurotoxic soluble waste products are continuously released from the NGVU, and the glymphatic pathway is, thus, considered a major route for the drainage of these toxic metabolites [3].

Unlike a more complete description of the glymphatic system in rodent brains [42], the existence of the glymphatic system in the human brain remains contentious as extensively reviewed by [25]. Based on the glymphatic pathway of the waste clearance process in rodent brains, three serial steps were recognised. First, the CSF (in bulk-flow) is repeatedly transported from the basal cistern into the subarachnoid space, then entering the peri-arterial spaces or PVS. Then, from the PVS, the CSF is transported into the ISF space through aquaporin 4 (AQP4) of the astrocytic end-feet processes that lead to CSF–ISF mixing and the removal of waste solute. Lastly, the CSF–ISF and interstitial waste solute mixture is then transported towards the peri-venous compartment of the larger central veins before exiting into the extracranial lymphatic vessels and systemic circulation [3,42]. 

The dysfunction of the glymphatic system has been speculated to be related to changes in the NGVU structure, particularly the PVS, whereby the enlarged PVS (ePVS) has been proposed to cause a glymphatic aberration that results in the accumulations of toxic metabolic products that are harmful to the brain microenvironment [43,44]. Moreover, AQP4 also plays an important rheological role in regulating the influx and efflux of ISF flow [45]. Several studies have shown that gene deletion and the reduced polarized expression of AQP4 on the astrocytic end-feet lining the PVS significantly reduced the glymphatic clearance, notably leading to the reduced clearance of amyloid beta (Aβ) in Alzheimer’s disease [42,46,47]. Moreover, aberrations in the glymphatic CSF–ISF exchange have been associated with various neuropathological conditions such as Alzheimer’s disease [48], multiple sclerosis [49], stroke [50], migraine with aura [51], traumatic brain injury [52], and even depression [53]. A recent animal study by the authors of [45] involving spontaneously hypertensive rats (SHRs), also demonstrated that an aberration in the glymphatic transport is implicated in the pathogenesis of arteriolosclerotic CSVD because of a combination of ePVS and a reduced astrocytic AQP4 polarity. Thus, the presence of ePVS may well indicate an aberrant NGVU and glymphatic system rheological dynamics, and the potential pathophysiological clue to numerous neuropathological conditions.

## 3. Parkinsonism and Vascular Parkinsonism

In the case of neurodegenerative diseases, ePVS (i.e., in basal ganglia) and the manifestation of parkinsonism has been reported [54,55,56]. Although their clinical significance remains unclear, multiple studies have shown that ePVS related to NGVU aberration leans towards parkinsonism-mediated cognitive impairments in addition to the known motor symptoms [57,58,59,60,61]. Moreover, as mentioned in the previous section, a reduced glymphatic clearance of Aβ accumulation in Alzheimer’s disease has been widely demonstrated. Hence, it has been hypothesised that the brain’s soluble phase alpha (α)-synuclein may have similar dynamics, although this remains under-studied. Abnormal levels of α-synuclein have been reported in several neurodegenerative diseases suggesting that an accumulation of this protein is neurotoxic and may lead to neuronal degeneration [62,63]. Recent studies also showed that this protein may be excreted into the extracellular space, in addition to their accumulation and conformation changes within the neurons (i.e., the deterioration of dopaminergic neurons). The former lends support to the potential involvement of an NGVU–glymphatic aberration, i.e., the CSF–ISF flow-mediated pathomechanism in parkinsonism [64,65]. In this section, we further elaborate on parkinsonism, including the definition, risk factors, and classifications, with a specific focus on vascular parkinsonism (VaP). 

### 3.1. Parkinsonism: Characteristic and Classification

Parkinsonism is a clinical syndrome denoted by an inconsistent combination of tremor, rigidity, postural imbalance, and bradykinesia that can happen due to various aetiologies [7,66]. Idiopathic parkinsonism or Parkinson’s disease (IPD) is the most common cause of parkinsonism, affecting up to 1.0 to 2.0% of people above the age of 60 years [67]. Globally, IPD is one of the most rapidly growing neurological disorders, whereby the Global Burden of Disease Study estimated an increase from 6 million in 2015 to 12 million cases by the year 2040 [68]. The primary pathological features of IPD are the prominent loss of dopaminergic neurons in the substantia nigra pars compacta and the progressive accumulation of Lewy bodies and Lewy neurites, mainly with aggregated α-synuclein within the surviving dopaminergic neurons [69,70].

Clinically, IPD is characterised by cardinal motor manifestations, including tremor, postural imbalance, and bradykinesia. Apart from motor symptoms, many PD patients suffer from gut-related symptoms such as constipation years before the diagnosis of PD [71,72]. However, the aetiology of IPD remains elusive, and atypical forms of parkinsonism include neurodegenerative diseases such as multiple system atrophy, dementia with Lewy bodies, progressive supranuclear palsy, and corticobasal syndromes (parkinsonism plus syndromes), secondary to drugs, toxins, infections, vascular diseases, and brain tumours (atypical/secondary parkinsonism) [73,74]. Among these parkinsonian disorders, recent interests in VaP are largely due to its distinct aetiological entity of IPD. In this context, there are several neuroimaging studies that showed distinctive morphometric measures and the magnetic resonance parkinsonism index (MRPI) between VaP and IPD that aided the differential diagnosis for VaP and IPD [75,76,77,78].

### 3.2. Vascular Parkinsonism (VaP)

VaP accounts for about 4.4 to 12% of parkinsonism and 3 to 5% of post-mortem or autopsy studies of parkinsonism patients [6]. It is typically described as symmetrical lower-body parkinsonism and associated with the presence of vascular lesions on brain imaging, particularly white matter hyperintensities (WMHs) or multi-infarcts in the basal ganglia and subcortical regions that are reported to be more common in elderly individuals (>65 years) and in males [7,8,9]. This distinctive phenotype was first described as ‘arteriosclerotic parkinsonism’ in 1929 by MacDonald Critchley in elderly patients with hypertension, characterised by rigidity, masked-like facies, and gait disturbances mainly associated with multitudinous vascular injuries, i.e., haemorrhages, lacunar, gliosis, and perivascular haemorrhages in the basal ganglia [79]. This concept was initially not well accepted by clinicians until the late 1980s when FitzGerald and Jankovic showed pathological evidence of multiple deep subcortical lesions in VaP when compared with IPD cases [80], whereby they introduced a more established term, ‘vascular parkinsonism syndrome’ or vascular parkinsonism’. 

Subsequently, Zijlmans and colleagues argued for the first clinical diagnosis criteria for VaP from their clinicopathological correlations [81], although specific diagnostic criteria for VaP needed further delineation [7]. Several known cardiocerebrovascular risk factors such as advanced age, hypertension, hyperlipidaemia, heart disease, and type-2 diabetes mellitus are recognised to be related to VaP. In addition, Glass and colleagues had suggested that VaP is neuropathologically distinct from IPD with the apparent lack or absence of α-synuclein, neuritic plaques, Lewy bodies, proliferation of glial cells, and instead had accumulations of Aβ within infarcted tissues [82]. The substantia nigra showed only mild depigmentation, most likely due to age-related neurodegeneration, comparable with the findings from brains of age-matched subjects without IPD symptoms [82]. In addition, VaP was associated with more prevalent ischemic cerebrovascular diseases, i.e., stroke and CSVD [7,66,83]. 

At present, three subtypes of VaP have been characterised: mixed, acute/subacute, and occult subtypes [84]. The mixed subtype is mostly diagnosed through molecular imaging using specific biomarkers such as dopamine transporters [85]. The acute or sub-acute post-stroke VaP is usually responsive to dopaminergic treatment or interventions and typically presents with an asymmetric parkinsonism [85]. Thirdly, the most frequent subtype is the occult (insidious) VaP. This subtype presents with progressive parkinsonism with characteristics such as gait impairment, postural instability, pseudobulbar, corticospinal, urinary symptoms, and cognitive impairment symptoms and tends to be poorly responsive to dopaminergic drugs [9,84].

Clinically, VaP presents with heterogeneous manifestations that may be distinguished from IPD phenotypically and from neuroimaging. The neuroimaging findings of VaP has been concisely reviewed elsewhere [85]. The main motor manifestations of VaP are lower body parkinsonism, denoted by bilaterally symmetrical gait difficulties, postural instability, freezing, and high incidence of falls [66,86]. Gait disturbance was found to be the earliest symptom in 90% of VaP patients, accompanied by shuffling and postural instability and, eventually, falls in the advanced phase [80,87]. The classical resting tremor is often absent, although postural tremor was evident [83,88]. 

Furthermore, other clinical features such as pyramidal tract involvement, dementia, pseudobulbar palsy, incontinence, sleep problems, pain, and gastrointestinal disturbances were more frequently observed in VaP [66,83,86,89]. VaP patients tend to be unresponsive to dopaminergic medication (i.e., levodopa) unless there is involvement of the nigrostriatal pathway [8,66]. VaP may appear suddenly in cases of basal ganglia infarcts. However, for most, it shows slow progression with subtle bilateral symptoms due to the diffuse subcortical white matter (WM) lesions or CSVD [66,82].

#### VaP Pathomechanism

A recent emerging understanding of vascular-related pathomechanisms in VaP may facilitate the discovery of potential therapeutic targets that could prevent/treat these underlying vascular injuries. Multiple small vessel-related mechanisms (e.g., lipohyalinosis) involved in the processes underlying vascular injuries in VaP have been proposed [81,89]. Advanced age and chronic hypertension are the two most important factors for the developments of WMH lesions in VaP. Hypertension chiefly causes blood vessel narrowing, resulted in a reduced cBF, which leads to ischemia and/or hypoxia in the vulnerable white matter regions. Following that, hypoxia and/or ischemia initiate a cascade of inflammatory activities induced by hypoxia-inducible factor-1a (HIF-1α), leading to the production of the transcriptional factor nuclear factor-kappa, B (NF-κB), free radicals, and proteases, causing BBB disruption, myelin loss, and endothelial dysfunction [90,91].

Moreover, the ischemia and/or hypoxia condition may result in mitochondrial dysfunction and protein inhibition leading to an imbalance of the antioxidants and reactive oxygen species (ROS) ratio, resulting in oxidative injury to vascular ECs, glia, and neuronal cells. This in turn could impair vascular functioning and neurovascular coupling, both of which affect the efficacy of the cBF leading to white matter damage [92,93]. In parallel, hypertension also affects the glymphatic drainage system, mainly involving elements within the NGVU microenvironment (i.e., PVS and AQP4 water channels) which result in waste accumulation in the brain and neurotoxicity, further leading to VaP [94,95]. As such, VaP might share common pathomechanisms with CSVD, particularly resulting from NGVU aberrations.

## 4. Cerebral Small Vessel Disease (CSVD)

**Figure 2:**
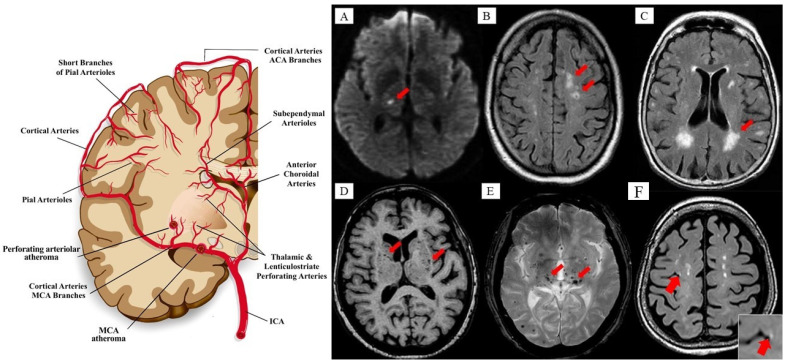
Vascular blood (Penetrating Arterial) supply to the brain, vasculo-pathological process (i.e., atheroma) of cerebral small vessel disease (CSVD), and neuroimaging manifestation and characterization of CSVD based on STandards for Reporting Vascular changes on nEuroimaging (STRIVE). (**A**) Diffusion-weighted imaging (DWI) showing recent small subcortical infarct (red arrow). Usual diameter is around 3–15 mm, with hyperintense rim surrounding ovoid cavity. It can also be seen as increased signal intensities in fluid-attenuated inverse recovery (FLAIR), T2-weighted, and DWI and decreased T1-weighted signal with isointense in T2*-weighted gradient-recoiled echo (GRE) signal and susceptibility-weighted imaging (SWI). It is best identified through DWI with usual infarct diameter of ≤20 mm. (**B**) FLAIR showing lacunar infracts (red arrow). Lacunar infarcts appeared as increased hyperintensity in T2-weighted signal, decreased T1-weighted, and FLAIR signal and isointense in DWI. Usual diameter is around 3–15 mm, with hyperintense rim surrounding ovoid cavity. (**C**) FLAIR showing white matter hyperintensities (WMHs) of presumed vascular origin (red arrow). WMHs seen as increased intensity or hyperintensity on T2-weighted imaging, T2*-weighted GRE and FLAIR (best identified), isointense on DWI, and hypointense (decreased intensity) on T1-weighted imaging. (**D**) T1-weighted imaging showing enlarged perivascular spaces (ePVS) (red arrow) with usual diameter of ≤2 mm. ePVS is seen as decreased FLAIR and T1-weighted signal intensity, with increased T2-weighted signal. Meanwhile, T2*-weighted GRE and DWI appeared isointense, and they also appeared in similar signal intensity with cerebrospinal fluid (CSF). (**E**) T2*-GRE showing cerebral microbleeds (CMBs) (red arrow). CMBs are small, rounded areas of signal void with blooming, whereby they were visualized as isointense T1- and T2-weighted signal, FLAIR, and DWI. They are best identified under T2*-weighted GRE or SWI as reduced signal intensities. Usual diameter is around ≤10 mm (mostly 2–5 mm). (**F**) T1-weighted (hypointense) of 3T MRI showing cortical microinfarcts (red arrow). Images (**A**–**E**), reproduced from [105], Frontiers, 2019, image F was adapted from [106], Mayo Clinic, 2019. ACA, anterior cerebral arteries; ICA, internal carotid artery; MCA, middle cerebral arteries.

Cerebral small vessel disease (CSVD) is a spectrum of chronic, progressive disorder affecting cerebral microvasculature (or microcirculation) involving small (50–400 µm) penetrating arteries, arterioles, capillaries, and small veins (or venules) that supply the white matter and deep grey matter of the brain subcortical region [4,5] (see Figure 2). CSVD is characterized by various clinical features and specific neuropathological and neuroimaging changes [96,97]. Moreover, CSVD is a dynamic disease process that is not limited to the cerebral vasculature alone but also involves other organ vasculature; hence, making it a heterogenous disease. It is reported as the most common cerebrovascular disease especially in the older population across the globe [96]. Its prevalence increases with aging and, thus, the silent (occult) or asymptomatic manifestation of CSVD is the most important determinant of dementia and stroke, accounting for almost 45% of vascular dementias globally, and one-fifth (20%) of all strokes, including 25% of ischemic strokes [97,98]. Interestingly, recent evidence had an increased incidence of VaP with CSVD [85,99]. 

Moreover, the advancement in various neuroimaging techniques has enabled the imaging-based identification and characterization of multiple manifestations of CSVD [100]. Neuroimaging, especially brain MRI (1.5 T to 7 T), has been widely utilized and serves as an in vivo visualization of brain tissue and blood vessel health, enabling the detection, characterization, and diagnosis of the wide spectrum of brain microcirculation disturbances. Vast arrays of MRI-based tools and techniques have been introduced with rapid evolution. However, the lack of standardization and consistency in these tools and techniques have led to the development of the STandards for Reporting Vascular changes on nEuroimaging (STRIVE), which have aided the image-based visual identification and classification of the CSVD spectrum [101] (see Figure 2). Several well characterized neuroimaging markers for CSVD include WMHs of presumed vascular origin or leukoaraiosis, lacunes of presumed vascular origin (i.e., small subcortical infarcts and silent brain infarcts), ePVS, cortical microinfarcts, and cerebral microbleeds (CMBs) [98,102]. Alarmingly, the lesions can be occult (or silent) and the affected individual may be asymptomatic (or not have any clinical symptoms). Equally, or if not more significant, these silent lesions, with a higher number of single or multiple lesions, are associated with a higher risk of mild cognitive impairment, dementias, Alzheimer’s disease, full-blown stroke, and parkinsonism [103,104].

### 4.1. Clinical Features of CSVD

It has been reported that CSVD was found to be 6 to 10 times more frequent in the incidence of first-ever stroke in the United States [107]. These silent brain infarcts are the most common incidental findings on neuroimaging, particularly in older individuals (i.e., 25% in ≥80 years individuals) [108], and it has been estimated that about 10 silent brain changes occur in every single/one symptomatic stroke [109]. Moreover, all neuroimaging features of CSVD were reported to be interrelated and widely correlated with undesirable impacts on cognitive functions and, thus, contributing to variable degrees of cognitive decline [110,111]. Whilst ethnicity and gender with adjusted age also explained the variability of these imaging findings, some findings reported higher WMHs grade and volume in certain ethnic or racial minorities than the non-Hispanic white ethnic group [112]. In addition, WMHs were reported to be much higher in women than men, although the exact mechanism for this gender difference remains elusive [113]. 

Moreover, a previous study had reported that stroke-free elderly Hispanics and/or Latinos appeared to have silent brain infarcts (in 16% of individuals), especially in the subcortical region (82.9%) [114] and ePVS (48%) [115]. In other ethnic groups, one study had reported that the prevalence of WMHs in South Asians and Europeans was similar, although South Asian elderly individuals with known vascular risk factors were more likely to have higher WMHs [116]. Meanwhile, data from three Asian countries (Singapore, Hong Kong, and Korea) showed that elderly Asians with a higher CSVD burden had a greater cognitive decline [117]. Meanwhile, the Taizhou Imaging Study reported incidental findings of WMHs (10.68%), lacunes (26.69%), CMBs (18.51%), and ePVS (27.76%) in elderly Chinese with collective vascular risks [118]. Finally, a study among the Japanese population reported that most healthy adults with related vascular risk factors possessed mild to moderate ePVS, especially in the centrum semiovale and basal ganglia [119].

CSVD clinically presents with ischemic stroke and/or lacunar stroke, whereby [120] described that small area/s of ischemia occur because of microemboli within the proximal sections of cerebral perforating arterioles. Generally, the changes may disturb the thalamo-perforating branches extending from the posterior cerebral artery, lenticulostriate branches extending from the anterior and middle cerebral arteries, and paramedian branches extending from the basilar artery and affect the pons, thalamus, basal ganglia, and white matter [120]. More recently, [121] documented that lacunar stroke may progress into several clinical syndromes, the most common being pure motor stroke (about 50–70% cases: isolated, pure motor paralysis or paresis), whilst other clinical syndromes include sensorimotor stroke, pure sensory stroke, dysarthria clumsy hand syndrome, and ataxic hemiparesis [121,122].

On the other hand, the chronic clinical manifestation of CSVD is chiefly related to the gradual progressive cognitive decline or impairment (i.e., from mild cognitive impairment to subcortical dementia) [123,124]. The disruption and/or reduced cerebral white matter integrity due to CSVD has been reported to result in parkinsonism-like symptoms presenting with a dominant gait and postural disturbance, minimal tremor of the limbs, early and symmetrical involvement of the lower extremities, sphincter dysfunctions (mainly urgent tenesmus and urinary incontinence), pseudobulbar syndrome [125,126], and depression [127,128]. The gradual progression of these symptoms may result in an individual losing his or her independence, withdrawing from social life, and experiencing falls and injuries, and an increased risk of death [127].

### 4.2. Emerging Pathomechanism of CSVD

The integrity of the NGVU or the BBB is closely linked to the state of the microcirculation and neural microenvironment within the brain parenchyma. To date, various and intensive investigations have been carried out to study the mechanisms of interaction of the cerebral parenchyma with its surrounding glial cells and microvasculature [129]. Central to this, is the role played by the NGVU in maintaining brain health and plasticity (capacity to recover) from insults that may paradoxically initiate an undesirable pathologic cascade leading towards cerebrovascular and/or neurodegenerative diseases, including CSVD. In fact, despite the growing insights from histopathologic, epidemiologic, physiologic, and imaging studies, the precise underlying pathomechanisms of CSVD required further validation. Generally, it is well understood that the pathomechanism of cerebrovascular disease associated with hypertensive changes in the vascular contribute to the development of arteriosclerosis or lipohyalinosis (thickening and/or damage the wall of arterioles), fibrohyalinosis, and the occlusion of cerebral penetrating arteries [130]. Such pathologies are thought to link with the ePVS and proliferation of connective tissue fibres that, subsequently, result in the loss of vascular contractibility before progressing into vascular and/or microvascular sclerosis.

Furthermore, pathological changes related to CSVD can occur following the occlusion of a cerebral small vessel by age-related arteriolar tortuosity and venous collagenosis, demyelination, and loss of glial cells [131,132,133]. These changes may be compounded by inflammaging, a term that had been proposed recently to describe age-related, chronic sterile low-grade inflammation as long-term effects of physiological stimulation involving the immune system [134,135]. These chronic effects involved various molecular and cellular mechanisms such as mitochondria dysfunction, gut microbiota dysbiosis, meta-inflammation, immunosenescence, and cellular senescence. Thus, inflammaging may arguably involve several other factors related to DNA repair, proinflammatory cytokines release and stem cell aging [135].

The DNA damage-mediated signalling transductions would result in cell cycle ar-rest, damage repair, apoptosis, and cell death [136,137]. In addition, age-related senescence-associated secretory phenotype is also recognised to be linked to the activation of the Toll-like receptors (TLRs)-mediated cytokines induction and nuclear factor kappa-light-chain enhancer of activated B cells (NF-kB) that regulate many genes that encode proinflammatory cytokines [138]. Indeed, defects in autophagy and stem cell aging are involved in regulating inflammaging at both the transcriptional and posttranscriptional levels [139]. Recent evidence has also shown that stem cell aging (i.e., the activation of intracellular sensor NOD-, LRR- and pyrin domain-containing protein 3 (NLRP3) inflammasome) and cellular senescence (i.e., due to multiple stress signals and characterized by cell cycle arrest—measured as the increased activation of p16INK4a and p53) are mediated by epigenetic dysregulation [140,141,142]. These findings emphasize the role of epigenetic regulation in terminally differentiated cells and stem cells in the context of altered DNA methylation, changes in histone modifications, and synergistic relationships between epigenetics and metabolism in aging. Of note, inflammaging has also been suggested to interact with several cardiocerebrovascular risk factors (such as hypertension, obesity, and type-2 diabetes mellitus) which in turn lead to the decreased cBF and BBB disruption in CSVD [143].

In relation to cBF, the recent use of an arterial spin labelling (ASL)-based imaging enabled the correlation of the cBF values with a burden of the WMHs as a potential biomarker for vascular cognitive impairment in CSVD and dementia [144]. Moreover, BBB breakdown may further deteriorate by an accompanying increased in the deposition of a blood component such as platelets where their activation also contributes to the formation of microthrombi in arteriosclerosis and/or arteriolosclerosis [145]. In response to an inflammatory signal, damaged endothelium releases the von Willebrand factors (vWF); hence, increasing the capacity of platelet activation and binding to vWF. Activated platelets also elevate the synthesis of the soluble vasospastic substance such as thromboxane A_2_ or B_2_ and adenosine diphosphate (ADP); the synthesis is possible after platelet binding with plasma fibrinogen [146]. These substances elicit the platelets and platelets–monocytes aggregations which have been used as markers for the onset and progression of arteriosclerosis and/or arteriolosclerosis that contribute to the microthrombi formation leading to CSVD manifestation [146].

Collectively, these physiopathologic responses would elicit further damage to the cerebral parenchyma (i.e., axonal injury, neuronal apoptosis, demyelination, and oligodendrocyte damage) leading to clinical manifestations, and multifaceted neuroimaging findings, including silent (occult) brain infarcts [97]. Notwithstanding, much of the current therapeutic insights seem to emerge from the pathomechanism of sporadic CSVD, from the molecular and cellular consequences of several systemic dysregulations that include coagulopathy, elevated microthrombosis, increased cellular activation, inflammation, and oxidative stress; parts or all of these processes could result in cerebral parenchyma microstructural changes that are widely recognised to occur in CSVD, which are endothelial dysfunction, altered cBF, and the breakdown of the BBB [97,147]. These microstructural changes also involve part of the NGVU components whereby physiopathologic responses at this cellular level affect the cerebral microcirculation ECs where complex interactions and dynamics are known to exist with several triggering cellular stressors, such as coagulation factors, cell activation, oxidative stress, and inflammation. Consequently, these responses may lead to endothelial dysfunction as well as subsequent NGVU aberrations.

## 5. CSVD and VaP: Clinicopathological Correlates

The main pathological feature of VaP is subcortical WMHs (a known CSVD manifestation) typically distributed within the midbrain, thalamus, and basal ganglia [148]. VaP is widely accepted as a type of parkinsonism that occurs in the context of cerebrovascular disease, although, in some cases, VaP could be misdiagnosed as IPD due to similar clinical features and mixed neuropathologies [9,148]. Brains of clinically confirmed IPD patients had pathological changes indicative of cerebrovascular damage, especially multiple lacunar infarcts in 36% of cases [149]. Pathological findings observed in VaP include findings confirming CSVD manifestation (i.e., lacunar infarcts, WMHs, and ePVS). 

Additionally, small vessel changes (i.e., CSVD pathologies) such as gliosis, perivascular pallor, hyaline arteriolosclerosis, and especially ePVS were observed in VaP autopsies [149]. Moreover, small subcortical lesions were reported to be distributed predominantly in the basal ganglia and thalamus of VaP patients [81,89]. Table 1 summarises the main CSVD correlates that provide a plausible pathophysiological link to VaP based on the reported past and recent case series [66,80,81,82,83,88,89,148,150,151,152,153,154,155,156,157,158,159].

Moreover, to date, most MRI studies have pointed to the prominent presence of basal ganglionic and diffuse subcortical WM lesions in VaP patients which clinically differentiate them from IPD (Lewy body) [66,83,88,150]. Importantly, Glass et al., documented that CSVD is strongly associated with the severity of WMHs and lacunar infarcts in VaP patients presenting with both motor and non-motor manifestations [82]. Following that, Dunet et al., showed a higher volume of WMHs lesions in VaP compared to IPD [151]. Moreover, Swallow et al., utilised the MRI-based Scheltens’ visual rating scoring scale and correlated the scores with cognitive functions, motor impairment, functional disability, and mood between VaP and IPD patients [152]. They reported higher Scheltens’ scores together with a higher prevalence of WMHs among VaP patients and concluded that VaP are more closely related to CSVD than IPD [152]. 

In one recent study, the normal appearance of WM in the corpus callosum was shown to be impaired; a finding that correlated with gait and postural instability which is generally seen in VaP patients [153]. Another innovative study used the functional MRI (fMRI) and fractional amplitude of low-frequency fluctuation (fALFF) approach to show functional connectivity in CSVD in the presence of gait disturbance (as proxy for parkinsonism). A reduced fALFF was found in regions associated with frontoparietal network, sensorimotor network, and reduced connectivity in CSVD patients with gait disturbances [154]. The reduced connectivity between the two regions influenced the gait speed, whilst the reduced fALFF in one of those regions (notably, the supplementary motor area) was associated with cadence, suggesting the role of the supplementary motor area in gait regulation [154]. Nevertheless, motor impairments such as gait and balance problems are also common in older adults without VaP and are associated with the presence of WMHs [155,156], although they lack other parkinsonism features. 

In addition, basal ganglia infarct and WMHs can manifest as asymptomatic or silent (occult) lesions in individuals without any history of stroke, which is more common among the elderly with hypertension [157,158]. One recent case report found multiple neuroimaging manifestations of CSVD (i.e., lacunar infarct and WMHs) in an 84-year-old man who presented with parkinsonism symptoms, where the brain autopsy revealed an evidence occult (insidious) subtype of VaP [148]. Hence, they conclude that the occult subtype of VaP occurred due to damage in the extra-nigrostriatal grey or white matter vascular lesions that had compromised the neuronal (dopaminergic) connectivity leading to parkinsonism, with the absence of the more distinct focal nigrostriatal deficits that are known to occur with IPD [160]. 

Therefore, the pathomechanism of VaP remains obscure, and is reflected by the lack of successful treatment approaches. The presence of a bilateral diffuse subcortical WM lesion in VaP has been speculated to be linked with motor symptoms, especially gait disturbances underlined by a dysfunction of the thalamocortical pathway, whereby there is disruption to the interconnecting fibre tracts between the basal ganglia, thalamus, and motor cortex affecting the sensory–motor interaction in the cortex [8,9]. Taken together, WMHs (a manifestation of CSVD) could well represent a common crosstalk of an ageing process with VaP manifestations [9]. Hence, the evidence in the preceding sections calls for the need to unravel the rheological clue between CSVD and age-related VaP, where the dynamics within the NGVU–glymphatic clearance could be the underlying common pathophysiological link between CSVD and VaP.

## 6. CSVD and VaP: The Clue in the Aberrant NGVU Dynamics

There is an increasing research interest to critically identify the exact mechanism targeting the NGVU-mediated neurodegeneration as a therapeutic intervention to effectively nullify and mitigate the development and progression of CSVD and/or VaP. Moreover, mounting evidence is emerging that ascribes the onset of a primarily neuronal aetiology in the background of an initial vascular pathology (often aging-related), i.e., from the insidious, occult CSVD to that of VaP onset [161]. Furthermore, there is growing interest in the research on the aetiology of such conditions in linking NGVU-mediated neurodegeneration with immune-mediated (i.e., inflammation and coagulation) mechanisms of brain injury from early stage, as the underlying primary feature of disease pathomechanisms. On that note, NGVU dynamics may serve as a rheological clue for the interaction of the neurodegeneration, vascular, and immune-mediated mechanisms for the cerebral parenchymal injuries. Here, we posit overlapping mechanisms involving the NGVU and glymphatic rheological links between CSVD and VaP. 

Numerous studies have shown that ePVS in arteriolosclerotic CSVD may be due to ex vacuo enlargement secondary to the shrinkage of brain tissue after axonal loss and demyelination [162], aberrant ISF drainage route [163], increased BBB permeability [164], and inflammation [165,166]. More recently, researchers have been focusing on alterations in the NGVU and glymphatic system to account for the ePVS in CSVD [45]. Although studies in this area are still scarce, hints to the impaired glymphatic system clearance are linked to the CSVD-related cardiocerebrovascular risk factors such as arteriosclerosis, reduced vSMCs efficiency, vascular tortuosity, and Aβ accumulation in the vessel wall [167,168,169,170,171]. Hence, interruptions to the NGVU dynamics may lead to AQP4 polarization and PVS loss of function as well as an impairment of cerebrovascular pulsatility that facilitates the CSF flow through PVS [42,172] Several pre-clinical models also supported the possibility of NGVU aberration, i.e., with a reduced expression of AQP4 polarization, impaired glymphatic (influx and efflux) system, and ePVS in CSVD (i.e., spontaneous hypertensive rat model) even at an early age [45,173,174]. 

In addition, there exists arterial and/or arteriolar remodelling, including the loss of vSMCs from tunica media that thickens the vessel wall leading to the narrowing of the microvessel lumen in CSVD [175]. Such vasculature remodelling is aggravated by known cardiocerebrovascular risk factors such as hypertension, whereby the continuous stimulation of abnormal mechanical signals leads to NGVU dysfunction and the subsequent impairment of astrocytic function [176,177] that could lead to the reduction in astrocytes AQP4 polarization. Interestingly, Zhang and colleague’s autopsy study proved the presence of VaP in individuals with a history of hypertension and hyperlipidaemia [148] that may reflect the underlying aberrant NGVU dynamics as the potential rheological clue to VaP. 

Moreover, as highlighted in earlier sections, ECs and pericytes also serve critical roles in the NGVU, whereby a lower pericytes coverage in animal models has been reported to be associated with a higher flow of neurotoxic molecules across ECs; hence, breaching the BBB and triggering the inflammation and detrimental cascade of neurotoxic events [178]. In fact, a recent study suggested that pericytes migration from the vessel wall may lead to a series of pathological conditions such as diabetic microangiopathy [179]. This has been supported by another research that showed pericytes dropped out from the vessel wall following an increase in the BBB permeability [178]. Moreover, others had proposed that hypertension-mediated reduction in the arterial pulsation may also lead to glymphatic system abnormalities since arterial pulsation is an important driver of the CSF–ISF flow in the NGVU and glymphatic system [148,174,180]. Hence, aberrant dynamics and biomechanics related to systemic hypertension may result in altered rheological dynamics within the multicellular NGVU components (the ECs, pericytes, astrocytes dysfunction, and AQP4 abnormalities) from their intercellular interactions, leading to neuropathological manifestations.

In this context, an immune-mediated pathogenesis also shapes our understanding of the pathophysiological link between CSVD and VaP. Upon BBB breakdown, the T cells probably enter the cerebral parenchyma and activate the antigen presenting cells that are distributed in the PVS [181,182]. Subsequently, these cells induce the differentiation and proliferation of encephalitogenic T cells that release pro-inflammatory cytokines, hence, heightening the parenchymal invasion of other immune cells [182,183]. Corroboratively, the clinical manifestation of CSVD such as ventricular enlargement has been recognised in several pre-clinical studies which was linked to the CSF–ISF flow in the NGVU–glymphatic system [184,185]. Besides ventricular enlargement, WMHs even at early stages have also been associated with a higher ISF in addition to demyelination [186]. These findings may reflect the underlying dilation or enlargement of PVS that could reduce the BBB integrity and, hence, provide easier diffusions for CSF with neurotoxic molecules to enter the cerebral parenchyma. 

On the other hand, reduced expressions of astrocytic AQP4 and the presence of striatal glial cells proliferation have also been associated with the dopamine regulation [187]. A reduced expression of AQP4 was recently shown to result in the loss of dopaminergic neurons in the ventral tegmental area and substantia nigra [188]. These observations highlighting the dopamine-related impairments associated with AQP4 dysfunction, could well relate to the aberrant dynamics of the NGVU–glymphatic system [161]. Thus, it is plausible that the subsequent disruptions to the clearance of neurotoxic or unwanted waste products, from an initial and insidious vascular injury (that manifests as WMHs, ePVS and/or infarction in CSVD), and if continued to progress, could trigger further neurodegenerative insults (that later manifest as VaP). 

**Figure 3:**
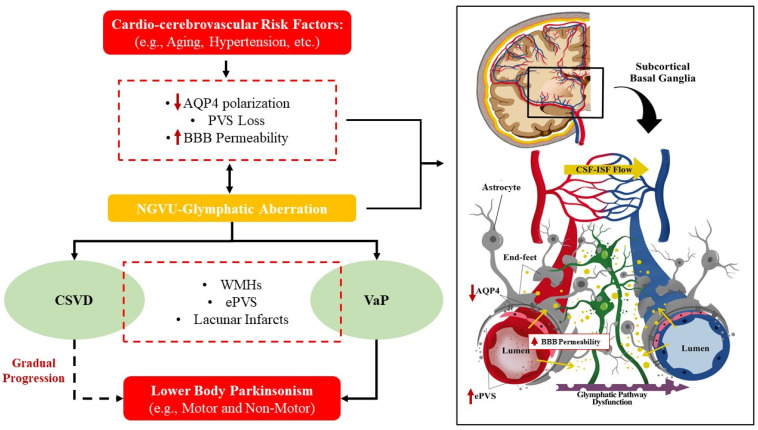
Proposition—cardiocerebrovascular risk factors may induce the aberration of neurogliovascular unit (NGVU) and glymphatic system dynamics (such as reduced astrocytic aquaporin 4 (AQP4) polarization, perivascular space (PVS) loss, and subsequent increase in blood–brain barrier (BBB) permeability (yellow arrow in left schematic diagram)—influence the transmigration of unwanted waste products—yellow dots in left schematic diagram) in cerebral small vessel disease (CSVD): a rheological clue to vascular parkinsonism (VaP). CSVD and VaP share similar neuroimaging manifestation seen as increased findings of white matter hyperintensities (WMHs), enlarged perivascular spaces (ePVS), and multiple lacunar or brain infarcts especially in subcortical regions (i.e., basal ganglia). These will eventually lead to clinical manifestation such as motor and non-motor lower body parkinsonism.

Taken together, the PVS loss and cell debris together with other waste products as a result of aberrant NGVU–glymphatic system dynamics leads to an impaired BBB, ePVS, cerebrovascular reactivity, immune-mediated inflammation, and impaired waste clearance from the CSF–ISF space forming the vicious loop of events within the neural microenvironment that lead to the pathogenesis of CSVD and subsequent VaP. The potential aberrant NGVU dynamics in CSVD that offer a putative rheological clue to VaP are summarized in Figure 3.

### Potential Pharmaceutics, Prophylaxis, Prevention, and Therapy for CSVD and VaP

The discovery and development of an effective treatment for CSVD and VaP remains the focus of ongoing research. The current advancement of neuroimaging techniques, especially MRI holds the feasibility to appraise any early lead to indicate an underlying endothelium dysfunction to prevent more deleterious consequences (i.e., BBB and glymphatic system dysfunctions) which could result in CSVD and VaP manifestations [189,190]. It has been proposed that advanced imaging tools to quantify imaging markers such as the WMHs volume, cBF, ePVS index, and cerebrovascular reactivity could facilitate a better and earlier detection of CSVD and, hence, facilitating the prevention/detection of VaP through more effective clinico-anatomical-guided therapeutic approaches [190]. Moreover, the use of molecular imaging techniques (i.e., dopaminergic transporter imaging) might be beneficial to better study the subtypes of VaP and CSVD co-morbidities [85]. Furthermore, in relation to the NGVU–glymphatic system, Navarro-Otano and colleagues measured that VaP had a normal to mild tracer uptake compared to IPD (using 123I-metaiodobenzylguanidine) using nuclear imaging technique such as SPECT imaging of striatal dopamine transporter [191]. Hence, the combination of MRI and SPECT may help in better diagnosing and segregating VaP from IPD.

To date, many VaP patients do not respond well to the typical dopaminergic medications used to treat IPD [9,99]. Hence, the treatment goal in VaP seems to focus more on lowering the chance of any future complications by controlling any known cardio-cerebrovascular risk factors in these patients. Additionally, as discussed, despite of their shared pathomechanics, both CSVD and VaP are heterogeneous disorders, medications (multitarget drugs or multidrug combination) and along with a neuropharmacological approach to treat the underlying risk factors, such as hypertension, hypercholesterolaemia, and diabetes mellitus, could provide viable therapies to prevent disease progression [192]. For instance, statin, a multi-targets drug (as anti-inflammatory, lowering cholesterol, and protecting endothelial cells) has been shown to attenuate the progression of WMHs, lacunes, and ePVS progression in CSVD patients (≥75 years) [193]. 

Moreover, healthy lifestyle practices such as eating a low fat–low salt diet, stopping smoking, and obtaining adequate physical exercise on a daily basis may be beneficial to reduce the risk of developing CSVD and its progression, and perhaps subsequent VaP and even future strokes. In fact, the therapeutic approaches to tackle obesity-linked inflammation would also address inflammaging [194], whilst other potential measures to mitigate inflammaging include the inhibition of NF-κB signalling to modulate the sensitivity of aged hematopoietic stem cells [195], inhibition of reverse transcriptase (using Lamivudine or natural compounds) [196], pharmacogenetic manipulation of p16INK4a-positive senescent cells to delay the aging onset [197,198], and modulating aging-associated inflammation and insulin resistance with a pattern recognition receptor (NLRP3) [199]. Last but not least, the white matter cerebrovascular damage as the pathophysiologic outcome from disruptions to NGVU–glymphatic clearance is recognised in the setting of both CSVD manifestations and incident VaP [125]. Others had even gone so far as to propose CSF drainage as a therapeutic approach for VaP [200,201,202,203], although a further clinical validation study is warranted.

## 7. Conclusions

The NGVU represents a rheological link for the dynamic interaction between brain injuries such as cerebrovascular, immune-mediated (inflammation, coagulation, and oxidative stress) and neurodegenerative mechanisms (i.e., with the onset/disease progression of CSVD and VaP). These pathomechanisms, it seems, revolve around the NGVU–glymphatic system or pathway of waste clearance, in which astrocytes and PVS are the key players in the setting of an aging-related insidious process over a lifetime that could inflict microenvironment damages that may lead to pathological conditions. Given the elusive interplay between these cerebrovascular-neurodegenerative ties, we present plausible rheological clues within the NGVU–glymphatic system as emerging neuroprotective targets that span the cerebrovascular and immune-mediated processes (inflammation, coagulation, and oxidative stress) to serve as a common CSVD-VaP therapeutic platform. Lastly, recent advancements in neuroimaging (MRI-based biomarkers) also offer the tools to further refine the role of this NGVU–glymphatic clearance dynamic for a mutually beneficial understanding in our continuing efforts to manage the two aging-related neurological conditions, CSVD and VaP.

## Figures and Tables

**Table 1:** CSVD correlates with VaP.

Risk Factors	Clinical Syndromes	Pathology	Neuroimaging Features
Advanced age Hypertension Stroke CSVD Hyperlipidemia Heart disease Diabetes mellitus Gender (Male > Female)	**Lower Body Parkinsonism:** **Motor** Symmetrical gait difficulties Postural instability Freezing Falls Postural tremor Pyramidal signs Pseudobulbar palsy Corticospinal signs **Non-motor** Urinary incontinence Dementia Sleep problems Pain Gastrointestinal disturbances	**CSVD** Gliosis Perivascular pallor Hyaline arteriolosclerosis ePVS **Small Recent Subcortical Lesions** Multiple lacunar infarcts (at Basal ganglia and Thalamus)	Diffuse subcortical WM lesions Basal ganglionic lesions

CSVD, cerebral small vessel disease; ePVS, enlarged perivascular spaces; VaP, vascular parkinsonism; WM, white matter.

## Data Availability

Not applicable.
